# Modulatory impact of *Bifidobacterium longum* subsp. *longum* BL21 on the gut–brain–ovary axis in polycystic ovary syndrome: insights into metabolic regulation, inflammation mitigation, and neuroprotection

**DOI:** 10.1128/msphere.00887-24

**Published:** 2025-02-03

**Authors:** Yao Dong, Shengnan Yang, Shu Zhang, Yuan Zhao, Xinlan Li, Mei Han, Zhonghui Gai, Kang Zou

**Affiliations:** 1 Germline Stem Cells and Microenvironment Lab, College of Animal Science and Technology, Nanjing Agricultural University, Nanjing, China; 2 Stem Cell Research and Translation Center, Nanjing Agricultural University 70578 , Nanjing, China; 3 Department of Food Science, Shanghai Business School 127231 , Shanghai, China; 4 Department of Research and Development, Wecare Probiotics Co., Ltd., Suzhou, China; University of Michigan-Ann Arbor, Ann Arbor, Michigan, USA

**Keywords:** polycystic ovarian syndrome, *Bifidobacterium longum *subsp. *longum*, gut–brain–ovary axis, inflammation mitigation, metabolic regulation

## Abstract

This study evaluates the efficacy of *Bifidobacterium longum* subsp. *longum* BL21 in mitigating symptoms of polycystic ovarian syndrome (PCOS) in DHT-induced PCOS model mice. It focuses on BL21’s role in modulating metabolic dysregulation, inflammation, and neuroprotection via the gut–brain–ovary axis. Employing an 8-week treatment regimen, this research assessed the effects of BL21 on prenatal androgen-induced PCOS in ICR mice. Evaluations included body weight, glucose tolerance tests, serum analyses of BDNF, inflammatory markers, sex hormone levels, and 16S rRNA gene sequencing for gut microbiota diversity and composition. Twenty-four ICR mice with induced PCOS served as subjects to examine the probiotic’s impact. Mice were administered a daily oral dose of 1 × 10^9^ CFU of BL21 continuously for a total of 8 weeks. BL21 significantly enhanced sex hormone levels (*P* < 0.05), particularly those of follicle-stimulating hormone (FSH) and estradiol (E2), indicating improved ovarian function and offering a novel PCOS treatment approach. The intervention notably curbed weight gain and improved glucose tolerance in PCOS mice (*P* < 0.05). BL21 reduced inflammatory markers such as interleukin-6 (IL-6), tumor necrosis factor-alpha (TNF-α), and lipopolysaccharides (LPS), while increasing IL-10, BDNF, FSH, and E2 levels (*P* < 0.05 for all). It also enriched gut microbiota diversity, enhancing populations of *Bifidobacterium* and *Lactobacillus*. Correlation analyses underscored the positive shifts in microbiota linked to beneficial hormonal and inflammatory profiles. BL21 shows promise in alleviating PCOS symptoms through metabolic regulation, inflammation reduction, and neuroprotection, validating its potential in integrated therapeutic strategies.

**IMPORTANCE:**

Polycystic ovarian syndrome (PCOS) is a prevalent endocrine disorder affecting women of reproductive age, characterized by metabolic irregularities, hormonal imbalances, and chronic inflammation. Existing treatments are often inadequate, addressing symptoms without targeting the underlying etiological factors. The investigation of *Bifidobacterium longum* subsp. *longum* BL21 as a probiotic intervention offers a novel approach by potentially regulating the gut–brain–ovary axis. This could lead to innovative therapeutic strategies that not only manage but also potentially reverse the multifaceted symptoms of PCOS, enhancing quality of life and reproductive health.

## INTRODUCTION

Medically, polycystic ovarian syndrome (PCOS) is a prevalent endocrine and metabolic disorder impacting women of reproductive age worldwide, with prevalence rates ranging from 6% to 20%. As a primary cause of female infertility, PCOS is closely associated with metabolic abnormalities such as insulin resistance, type 2 diabetes, obesity, and cardiovascular diseases, marking it as a critical public health issue (1–3). The diagnostic criteria for PCOS encompass ovulatory irregularities, hyperandrogenism, and polycystic ovarian morphology, as evidenced by ultrasound imaging (4). PCOS manifests heterogeneously, ranging from mild menstrual irregularities to severe metabolic complications, with early signs including menstrual acyclicity, anovulation, and weight gain, progressing to obesity, impaired glucose tolerance, dyslipidemia, and hepatic steatosis (5). The complexity positions PCOS at the nexus of endocrinology, reproductive medicine, metabolic disease, and psychology. Current therapeutic strategies aim to improve reproductive health and diminish long-term health risks, utilizing oral contraceptives, metformin, clomiphene, antiandrogens, and insulin sensitizers (2, 6). While these interventions can be effective in alleviating some aspects of PCOS, they do not address its underlying etiology and are associated with a range of potential side effects. Oral contraceptives may cause gastrointestinal issues, weight gain, and mood shifts. Metformin can lead to nausea and diarrhea, while antiandrogens, effective against hirsutism, may induce fatigue and are contraindicated during pregnancy. Moreover, these treatments can disrupt gut microbiota, exacerbating metabolic and inflammatory complications inherent to PCOS (7–9). This highlights the urgent need for comprehensive treatment strategies that improve quality of life and health outcomes for PCOS patients, emphasizing the critical role of gut microbiota in an integrated PCOS management approach.

Gut microbiota dysbiosis is increasingly recognized as a crucial factor in PCOS pathogenesis, contributing to metabolic disturbances and enhanced inflammatory responses that shape its clinical manifestations (10). The mechanisms through which dysbiosis influences PCOS are complex. Enhanced gut permeability associated with dysbiosis may facilitate the translocation of pro-inflammatory molecules such as lipopolysaccharides (LPS). These molecules contribute to systemic inflammation, further impairing insulin signaling and exacerbating hyperinsulinemia, a key driver of hyperandrogenism in PCOS (11, 12). Additionally, altered gut microbiota can disrupt the normal estrogen metabolism pathway, leading to an imbalance in estrogen and androgen levels, which are critical in the pathophysiology of PCOS (13, 14). Metabolic dysfunctions such as insulin resistance, obesity, and glucose dysregulation are frequently observed with PCOS in clinical settings (15), while inflammatory processes exacerbate symptoms like irregular ovulation and hirsutism. Chronic inflammation, indicated by elevated interleukin-6 (IL-6) and LPS levels, may directly affect ovarian function and androgen metabolism (16, 17). Additionally, the gut–brain axis, highlighting the interplay between intestinal microbiota and brain, significantly influences the mental health of PCOS patients. This axis serves as a critical communication pathway, where alterations in gut microbiota may impact neurological and hormonal functions essential for the regulation of ovarian function, known as the gut–brain–ovary axis. Dysbiosis within this pathway can precipitate neuroendocrine changes that are implicated in the psychological symptoms frequently observed in PCOS patients, such as anxiety and depression. These psychological disturbances, in turn, may influence ovarian function and disrupt hormonal balance, exacerbating PCOS symptoms (18). Recent studies advocate probiotics as a promising treatment, showing therapeutic benefits in improving reproductive cycles, reducing androgen synthesis, and restoring gut microbiota diversity in PCOS models (19). Specifically, probiotics such as *Bifidobacterium* and *Lactobacillus* have been effective in adjusting metabolic and sex hormonal imbalances, mitigating inflammation, and enhancing gut microbiota richness and diversity (19). *Bifidobacterium longum* subsp. *longum* BL21, in particular, has demonstrated efficacy in correcting metabolic abnormalities induced by high-fat diets (20), managing type 2 diabetes via gut microbiota modulation (21), and highlighting its potential in liver–gut axis regulation (22). These findings collectively suggest BL21’s potential as a valuable component in PCOS treatment strategies, offering a novel approach to modulate intestinal microbiota, metabolic pathways, and inflammatory responses, thereby alleviating PCOS symptoms.

In this study, a dihydrotestosterone (DHT)-induced PCOS mouse model was used to mimic the reproductive and metabolic dysfunctions associated with PCOS (23). DHT, a potent androgen, is derived from testosterone through the catalytic action of 5α-reductase and notably does not convert to estrogen (23). The use of DHT in mouse models, particularly during critical developmental stages such as pregnancy and adolescence, is a common practice in scientific investigations (24). The DHT-induced model has successfully replicated numerous key aspects of human PCOS, including alterations in the reproductive system, obesity and weight gain, metabolic dysregulation, dyslipidemia, endocrine imbalances, and elevated inflammatory markers (19). Using this model, we evaluated the potential therapeutic effect of the strain BL21 in alleviating the symptom of PCOS by administering BL21 as an intervention treatment. This evaluation encompassed the probiotic’s impact on metabolic levels, immune modulation, and diversity of the gut microbiota, in addition to exploring its underlying mechanisms in mitigating PCOS symptoms, and the result showed a beneficial outcome in alleviation of PCOS, which is helpful to develop novel therapeutic strategies for PCOS in clinic, thereby enhancing the array of treatment options available for this multifaceted disorder.

## RESULTS

### Phenotypic validation of the prenatal androgen-induced PCOS model

**Fig 1:**
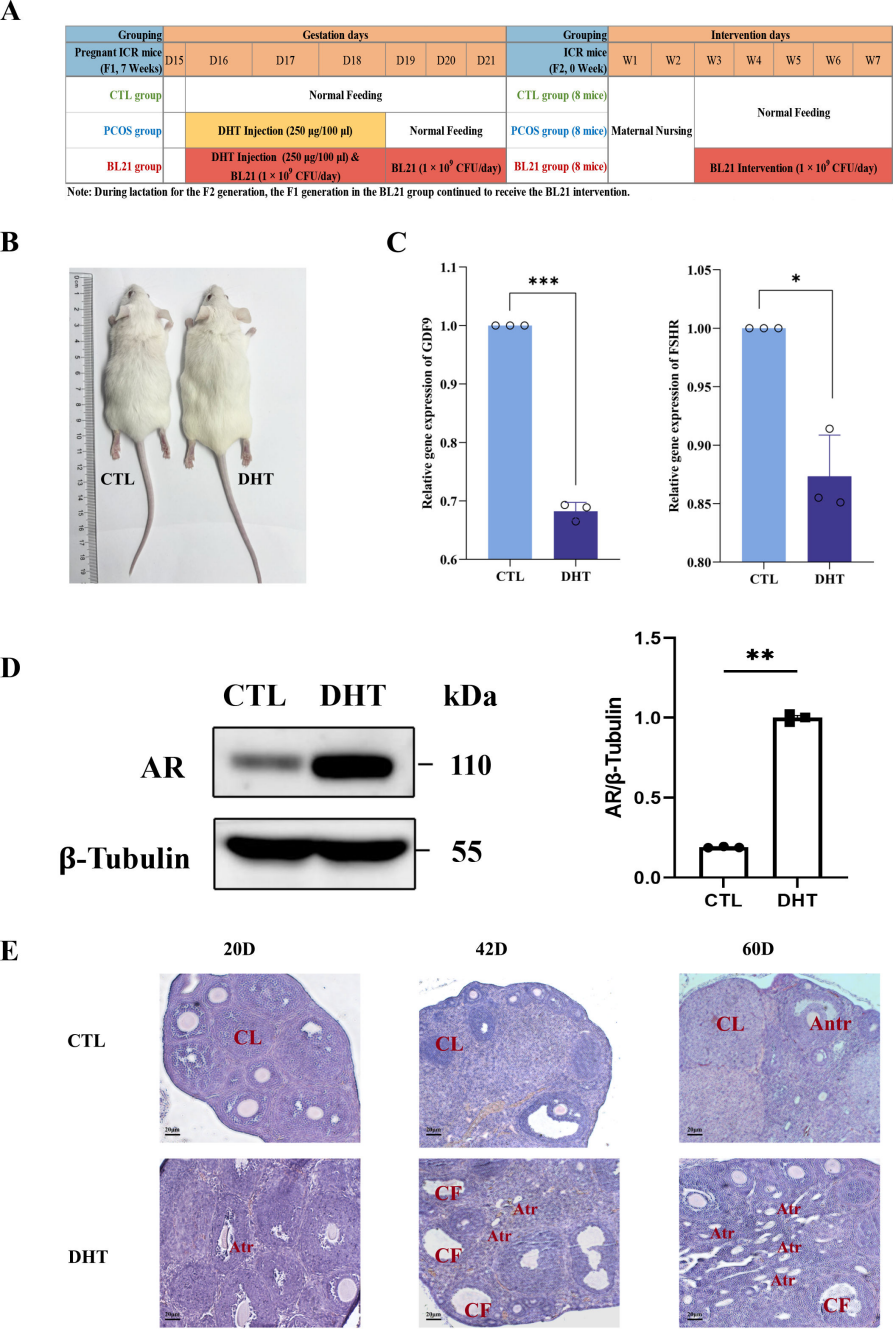
Establishment of the polycystic ovarian syndrome (PCOS) model and effects of *Bifidobacterium longum* subsp. *longum* BL21. (**A**) Intervention process for BL21 in the PCOS mouse model; (**B**) body phenotype comparison on day 60 between the CTL and dihydrotestosterone (DHT)-induced PCOS mice; (**C**) relative gene expression levels of growth differentiation factor 9 (GDF9) and follicle-stimulating hormone receptor (FSHR) in the ovaries; (**D**) Western blot analysis of androgen receptor (AR) expression in ovaries; (**E**) hematoxylin and eosin (H&E)-stained ovarian tissue sections from mice aged 20, 42, and 60 days, showing the microstructure of the ovaries at different developmental stages. Annotations include the corpus luteum (CL), cystic follicles (CF), atretic follicles (Atr), and antral follicles (Antr), demonstrating changes in the ovarian structure. Statistical significance compared to the control (CTL) group is indicated by *P < 0.05, **P < 0.01, and ***P < 0.001.

At 42 days of age, PCOS model mice showed significant phenotypic differences compared to normal controls, with DHT treatment leading to pronounced obesity and substantial abdominal adiposity accumulation (Fig. 1B). Gene expression analysis in oocytes and granulosa cells from the DHT-treated mice revealed a marked reduction (Fig. 1C). In addition to the phenotypic changes observed, Western blot analysis performed to validate the feasibility of the prenatal androgen-induced PCOS model further substantiated the pathological impact of DHT treatment. Figure 1D illustrates a significant upregulation of androgen receptor (AR) expression in the ovaries of DHT-treated mice compared to controls. The increased AR protein level was significantly higher in the PCOS group, confirming enhanced androgen signaling. Densitometric analysis quantified this increase, with AR levels normalized to β-tubulin showing a robust enhancement (*P* < 0.01) in response to DHT treatment. The elevation in AR expression serves as a critical molecular marker, validating the DHT-induced model’s ability to mimic the hormonal dynamics central to PCOS pathogenesis. Moreover, a detailed examination of ovaries at 20, 42, and 60 days highlighted progressive changes: initial sporadic atretic follicles at 20 days, increased cystic follicles with sexual maturity at 42 days characterized by enlarged cavities and reduced granulosa cell layers, and a higher prevalence of atretic follicles by 60 days. In contrast, control mice consistently exhibited healthy follicles, corpora lutea, and dense granulosa cell layers across all time points (Fig. 1E).

### Effects of BL21 on body weight, glucose tolerance, and serum biomarkers in the PCOS mouse model

**Fig 2:**
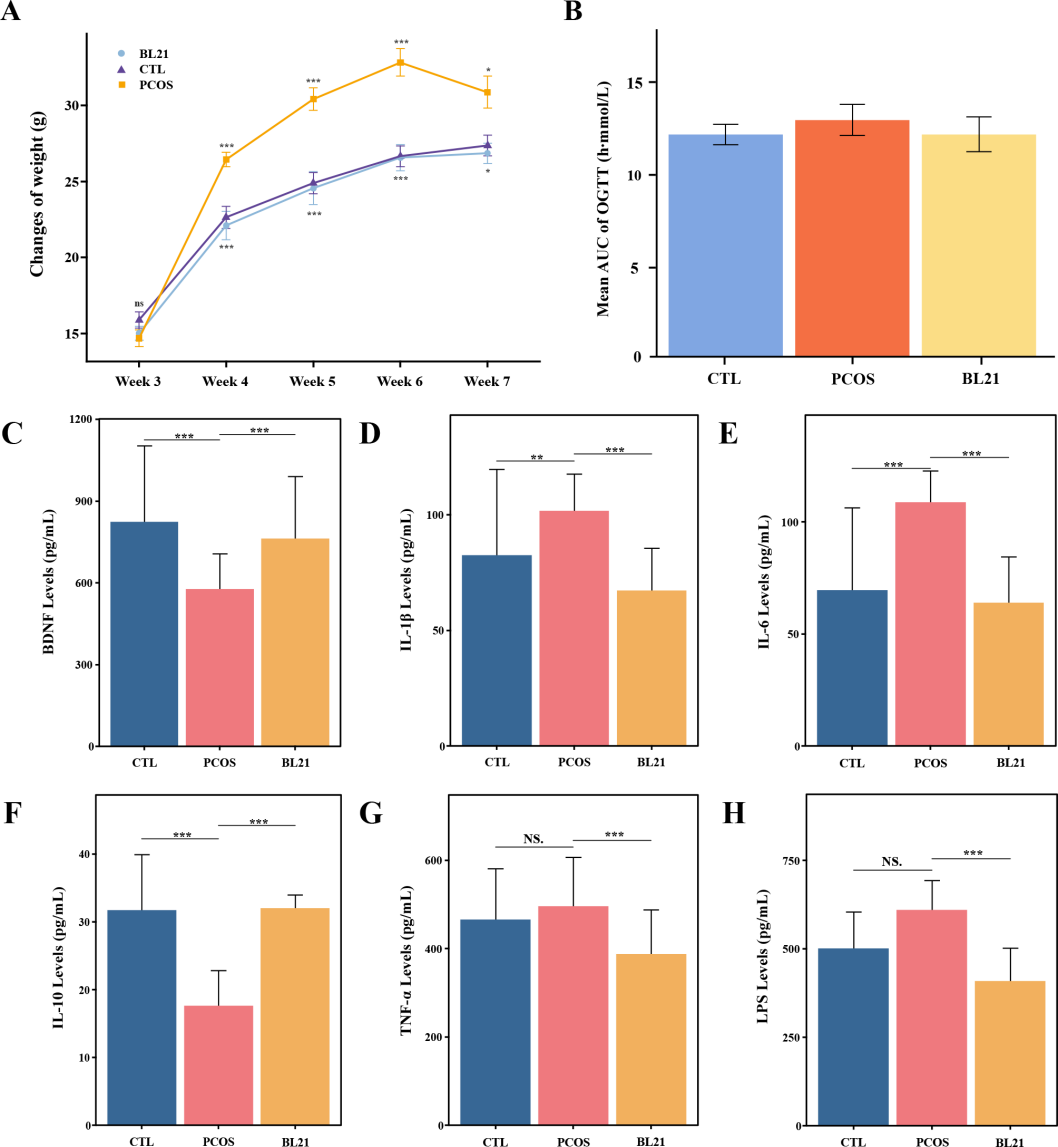
Effects of *Bifidobacterium longum* subsp. *longum* BL21 on body weight, oral glucose tolerance, and serum cytokines in PCOS mice. (**A**) Mouse body weight changes; (**B**) results of the OGTT, represented by the area under the curve (AUC), demonstrating the effect of BL21 intervention on glucose metabolism in PCOS mice; (**C–H**) serum biomarker analysis, including brain-derived neurotrophic factor (BDNF) (**C**), interleukin-1β (IL-1β) (**D**), interleukin-6 (IL-6) (**E**), interleukin-10 (IL-10) (**F**), tumor necrosis factor-α (TNF-α) (**G**), and lipopolysaccharides (LPS) (**H**). Statistical significance compared to the CTL group is denoted by *P < 0.05, **P < 0.01, and ***P < 0.001, with NS indicating not significant (*P* > 0.05).

During the study, body weight trends depicted in Fig. 2A showed no significant differences among groups in the first 3 weeks. From week 4, the PCOS model group exhibited a significant increase in weight gain compared to the CTL group (*P* < 0.05). Notably, the weight gain trajectory of the PCOS model mice subjected to the BL21 intervention mirrored that of the CTL group, exhibiting a significant difference from the PCOS group (*P* < 0.05). Oral glucose tolerance test (OGTT) results (Fig. 2B) revealed impaired glucose tolerance in the PCOS group, with a higher glucose AUC than the CTL group. BL21 treatment improved glucose AUC values toward those of the CTL group. Serum cytokine analysis showed reduced BDNF and IL-10 levels in the PCOS group compared to the CTL group (*P* < 0.001), which BL21 intervention significantly increased, nearing normative levels (Fig. 2C and F). Pro-inflammatory cytokines IL-1β and IL-6 were significantly higher in the PCOS group than in the CTL group (*P* < 0.01; Fig. 2D and E), suggesting a chronic inflammatory state in PCOS. TNF-α and LPS levels were elevated in the PCOS group but not significantly; BL21 treatment significantly reduced these levels (*P* < 0.001; Fig. 2G and H).

### Effects of BL21 on sex hormone levels, ovarian morphology, and AR expression in a PCOS mouse model

**Fig 3:**
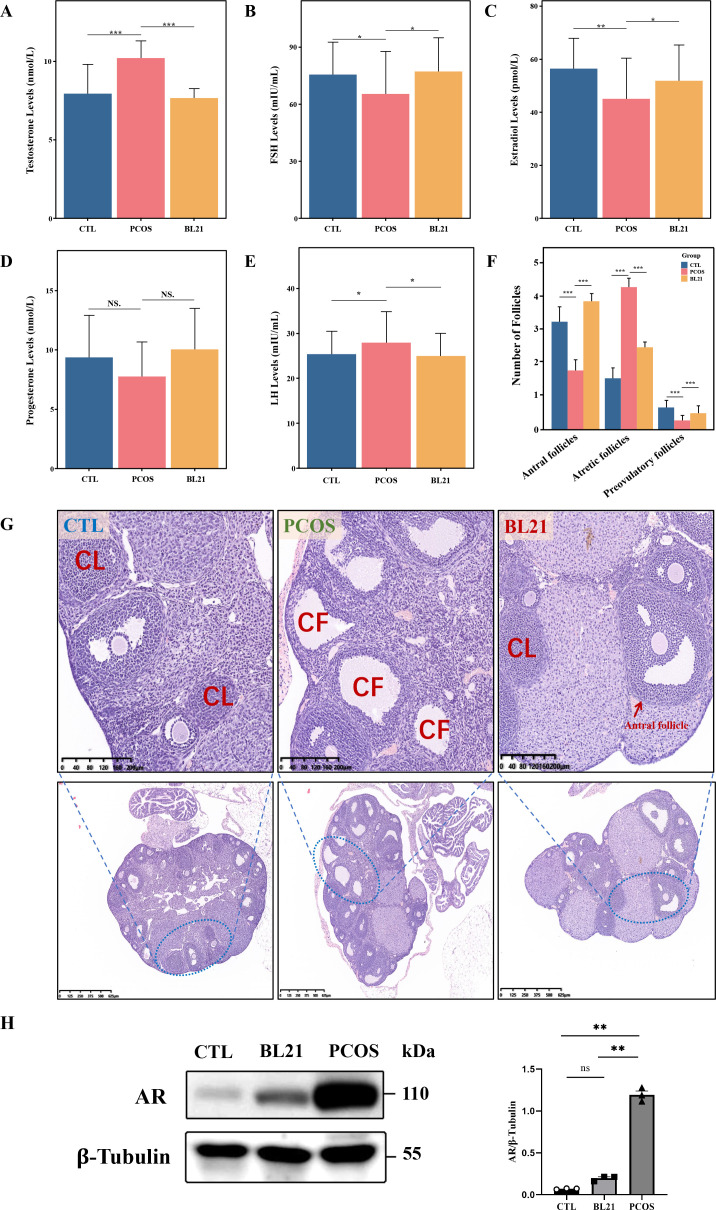
Impact of *Bifidobacterium longum* subsp. *longum* BL21 on sex hormone levels and ovarian morphology in PCOS mice. (**A–E**) Serum sex hormone level analysis: (**A**) testosterone; (**B**) follicle-stimulating hormone (FSH); (**C**) estradiol (E2); (**D**) progesterone (PROG); (**E**) luteinizing hormone (LH); (**F**) quantification of ovarian follicles, including antral, preovulatory, and atretic follicles, based on eight independent samples (*n* = 8), showing the effect of BL21 intervention on follicle development and count; (**G**) H&E-stained sections of ovarian morphology, highlighting the corpus luteum (CL) and cystic follicles (CF) and demonstrating the improvement in ovarian morphology following BL21 intervention; (**H**) Western blot analysis of androgen receptor (AR) expression in CTL, BL21, and PCOS groups. Statistical significance compared to the CTL group is indicated by *P < 0.05, **P < 0.01, and ***P < 0.001, with NS indicating nonsignificant (*P* > 0.05).

In the PCOS mouse model, serum analysis revealed significant sex hormonal imbalances compared to controls. Testosterone and LH levels were markedly higher (*P* < 0.001 and *P* < 0.01, respectively), whereas FSH and E2 levels were significantly lower (*P* < 0.05 and *P* < 0.01, respectively), with a slight, nonsignificant drop in PROG. BL21 treatment notably decreased testosterone and LH (*P* < 0.001 and *P* < 0.05, respectively) and significantly raised FSH and E2 levels (both *P* < 0.05), with a nonsignificant increase in PROG (Fig. 3A- E). Follicular analysis (Fig. 3F) showed that PCOS model mice had increased atretic follicles and fewer antral and preovulatory follicles (*P* < 0.001). Post-BL21 intervention, there was a significant reduction in atretic follicles and an increase in antral and preovulatory follicles, indicating improved follicular quality. Ovarian morphology also improved, with fewer cystic follicles observed after BL21 treatment (Fig. 3G). Moreover, analysis of AR expression showed an increase in AR levels in the PCOS model, which was significantly mitigated by BL21 treatment (Fig. 3H). These results highlight BL21’s potential in correcting sex hormonal imbalances and improving ovarian morphology in PCOS.

### Effects of BL21 on the gut microbiota diversity and structure in a PCOS mouse model

**Fig 4:**
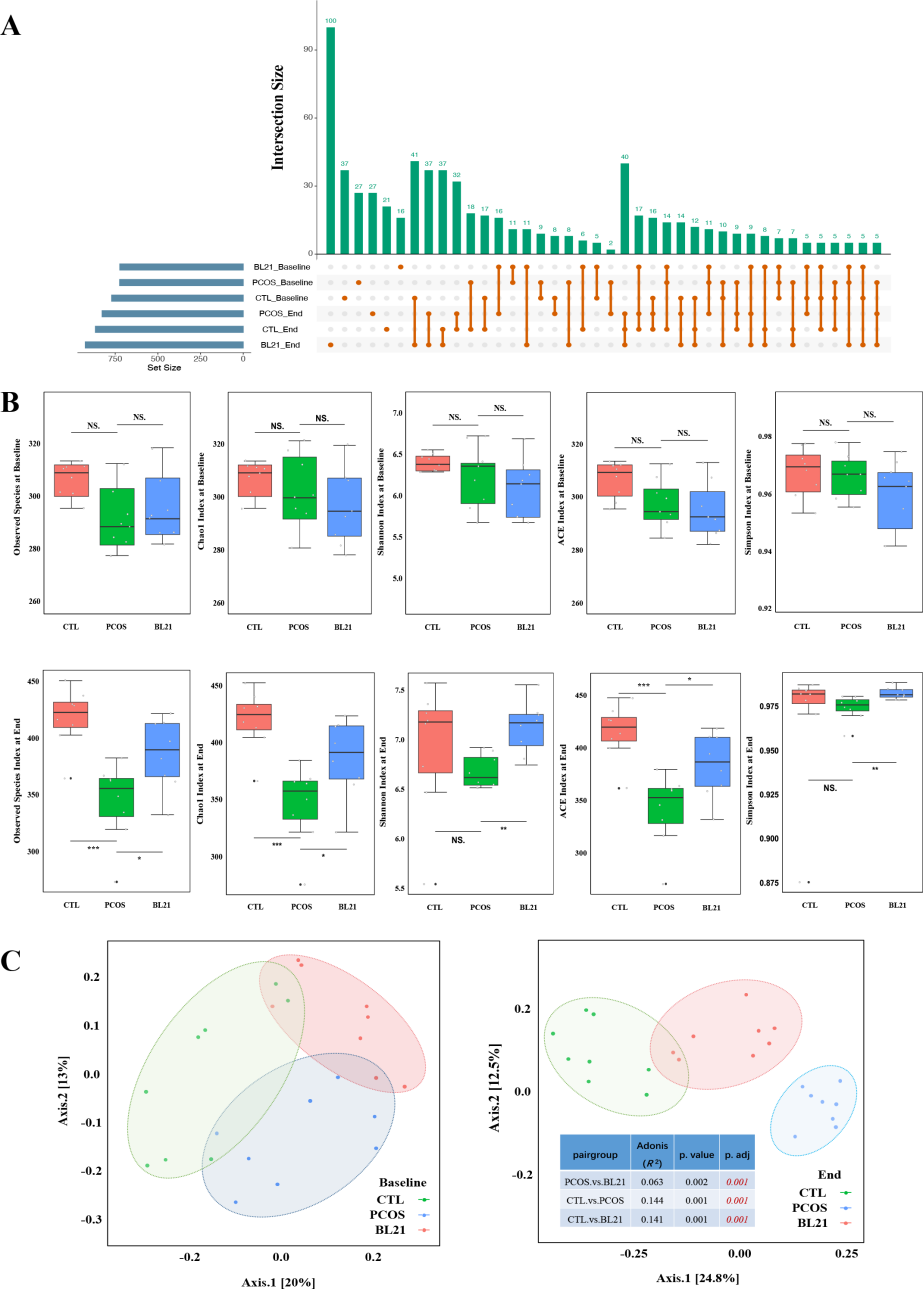
Influence of *Bifidobacterium longum* subsp. *longum* BL21 on gut microbiota diversity in PCOS mice. (**A**) UpSet plot showing the composition and accumulation of microbial species across different experimental groups of mice; (**B**) box plot of alpha diversity analysis pre- and post-BL21 intervention, showing changes in gut microbiota diversity. The box represents the interquartile range, and the line inside the box denotes the median. Statistical significance compared to the CTL group is marked by *P < 0.05, **P < 0.01, and ***P < 0.001, with NS indicating not significant (*P* > 0.05). (**C**) Beta diversity analysis using principal coordinates analysis (PCoA) to demonstrate changes in gut microbiota structure pre- and post-BL21 intervention. Adonis analysis assesses the significance of differences in beta diversity between groups.

Using an UpSet plot, we visually analyzed data set group intersections before and after BL21 intervention (Fig. 4A), observing minimal initial differences among CTL, PCOS, and BL21 groups. Post-intervention, the BL21 group showed a significant increase in gut microbiota species count, affecting 100 specific species, compared to 21 and 27 species in CTL and PCOS groups, respectively, highlighting BL21’s impact on gut microbiota diversity in PCOS mice. Alpha diversity analysis (Fig. 4B) revealed no initial differences across groups in diversity indices (*P* > 0.05). Post-intervention, the PCOS group showed significant reductions in diversity indices compared to CTL, while the BL21 group exhibited significant improvements in all indices versus PCOS (*P* < 0.05), suggesting BL21 restores gut microbiota diversity to a normative level. Beta diversity analysis (Fig. 4C) using Bray–Curtis distances and NMDS showed baseline microbiota structure clustering among groups became dispersed post-intervention, indicating significant microbiota structural changes. PCoA confirmed these findings, underscoring BL21’s substantial effect on gut microbial community structure.

**Fig 5:**
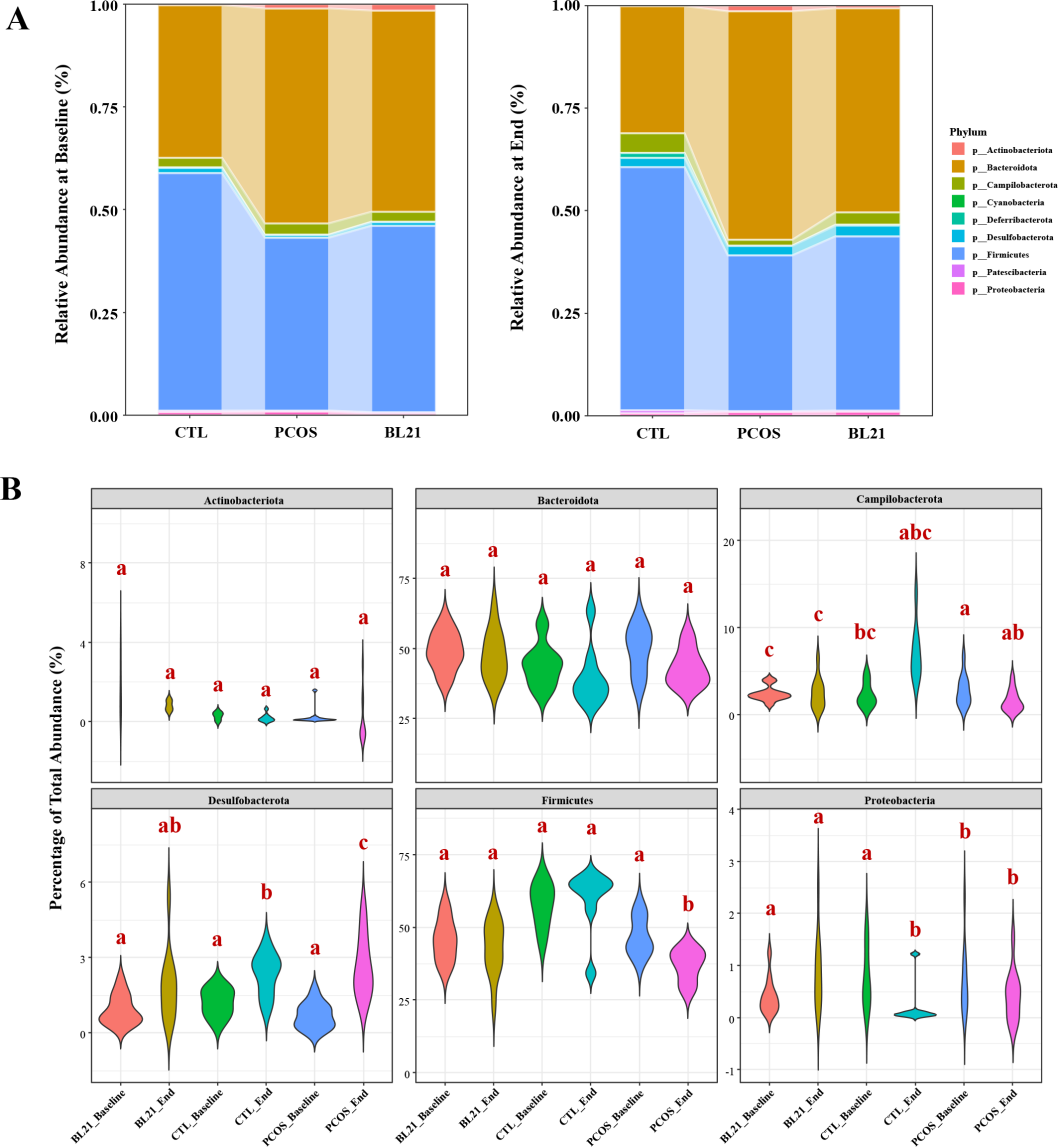
Analysis of the impact of *Bifidobacterium longum* subsp. *longum* BL21 on mouse gut microbial communities at the phylum level. (**A**) Cumulative graph showing changes in the abundance of gut microbiota at the phylum level before and after treatment with BL21. (**B**) Violin plots illustrating differences in gut microbiota abundance at the phylum level before and after BL21 intervention at different time points. Different letters indicate statistically significant differences between groups.

Analysis presented in Fig. 5A reveals that despite variability, Bacteroidota and Firmicutes were the dominant phyla across all groups. Post-intervention analysis (Fig. 5B) showed no significant differences in Actinobacteriota and Bacteroidota abundances between BL21 and PCOS groups. However, significant differences in Campilobacterota, Desulfobacterota, Firmicutes, and Proteobacteria abundances were observed between BL21 and PCOS groups (*P* < 0.05). Compared to CTL, the PCOS group exhibited no significant changes in Actinobacteriota, Bacteroidota, Campilobacterota, and Proteobacteria, but significant differences in Desulfobacterota and Firmicutes were observed (*P* < 0.05). These results highlight BL21’s significant effect on specific bacterial phyla abundances.

**Fig 6:**
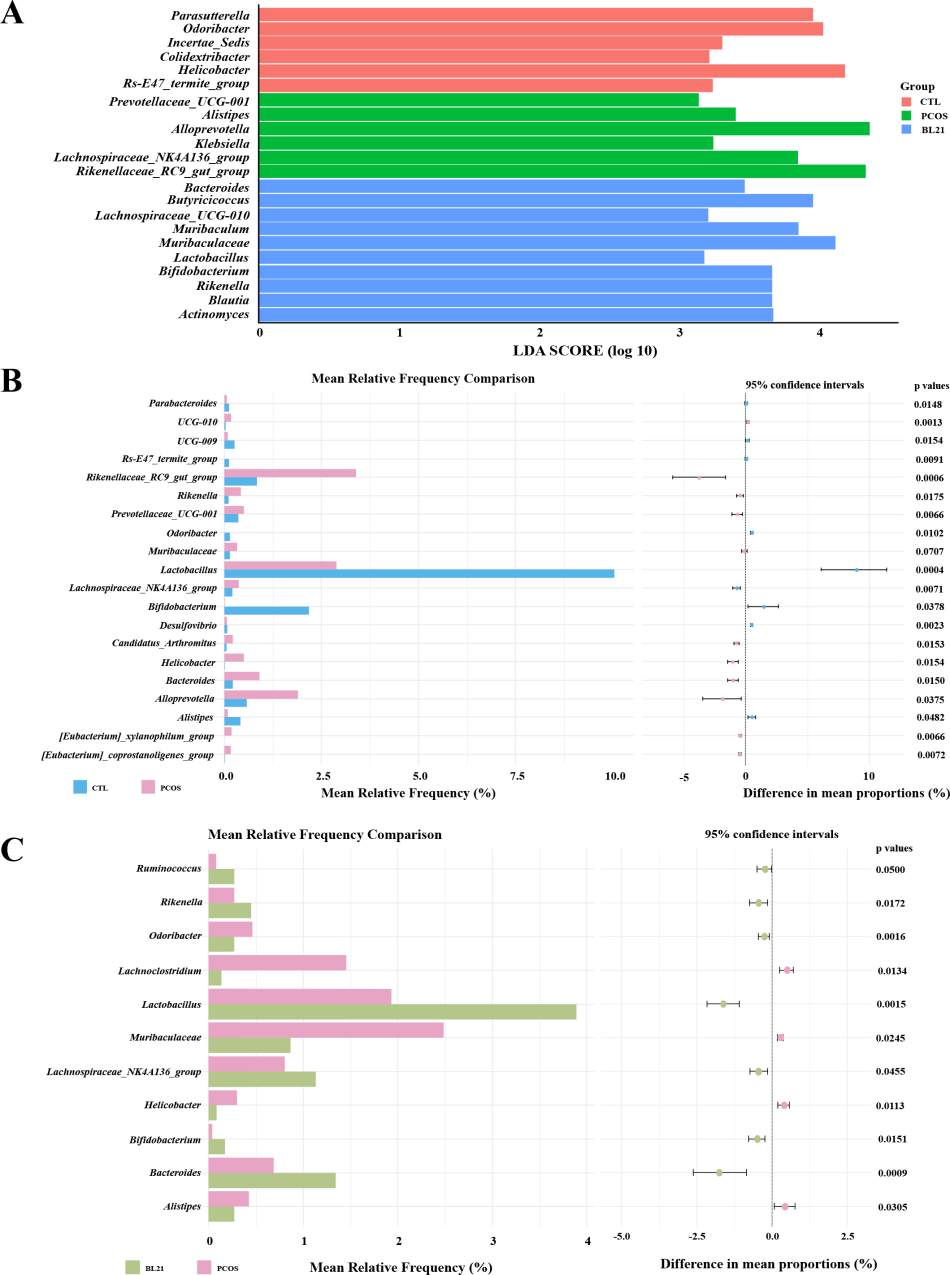
The impact of *Bifidobacterium longum* subsp. *longum* BL21 on mouse gut microbiota at the genus level. (**A**) Results of the linear discriminant analysis effect size (LEfSe) analysis, showing the impact of BL21 treatment on differences in the abundance of bacterial taxa at the genus level. LEfSe analysis evaluates each significantly enriched bacterial taxonomic unit by calculating linear discriminant analysis (LDA) scores; (**B**) LEfSe analysis results at the genus level between the DHT-induced PCOS mouse model and the control (CTL) group; (**C**) post-intervention LEfSe analysis results at the genus level between the DHT-induced PCOS mouse model and the BL21 treatment group.

Utilizing linear discriminant analysis effect size (LEfSe) and STAMP, this study identified significant genus-level differences in gut microbiota across experimental groups. LEfSe analysis showed the BL21 group had increased abundance of beneficial genera such as *Bifidobacterium*, *Butyricicoccus*, *Lactobacillus*, and *Blautia* compared to CTL and PCOS groups (Fig. 6A). STAMP analysis further detailed genus-level differences, revealing a decrease in *Lactobacillus*, *Parabacteroides*, and *Bifidobacterium* in the PCOS compared to CTL groups, with increases in *Helicobacter*, *Alloprevotella*, and *Rikenellaceae_RC9_gut_group* (Fig. 6B). BL21 intervention countered PCOS-induced shifts, reducing the abundance of *Helicobacter*, *Odoribacter*, *Alistipes*, and *Muribaculaceae* and boosting that of beneficial genera like *Lactobacillus*, *Bifidobacterium*, *Bacteroides*, and *Ruminococcus* compared to PCOS (Fig. 6C).

### Analysis of the correlations between gut microbiota and inflammatory cytokines, sex hormones, and BDNF

**Fig 7:**
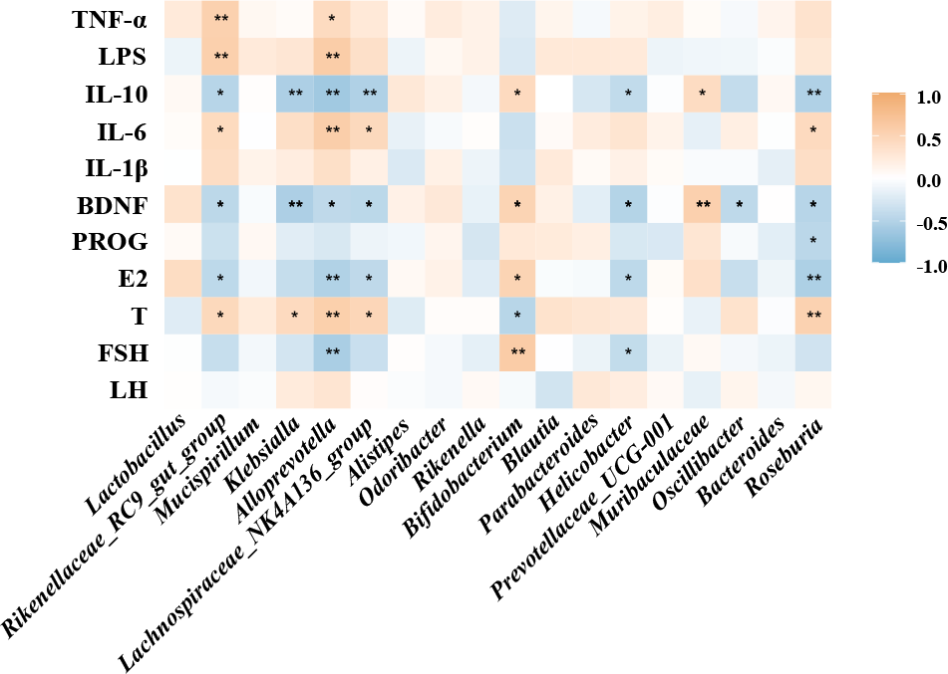
Correlation analysis between gut microbiota and inflammatory cytokines, sex hormones, and brain-derived neurotrophic factor (BDNF). This figure presents the correlation analysis results between gut microbiota and the following inflammatory cytokines, sex hormones, and BDNF: tumor necrosis factor-alpha (TNF-α), interleukin-6 (IL-6), interleukin-10 (IL-10), interleukin-1beta (IL-1β), lipopolysaccharides (LPS), brain-derived neurotrophic factor (BDNF), progesterone (PROG), estradiol (E2), testosterone (T), follicle-stimulating hormone (FSH), and luteinizing hormone (LH).

Correlation analysis revealed complex relationships between gut microbiota and inflammatory cytokines, sex hormones, and BDNF (Fig. 7). In PCOS-treated mice, genera like *Alloprevotella*, *Klebsiella*, and the *Lachnospiraceae_NK4A136_group* correlated positively with markers (T, TNF-α, IL-6, and LPS) and negatively with FSH, E2, BDNF, and IL-10. Conversely, in BL21-treated mice, beneficial genera such as *Bifidobacterium*, *Bacteroides*, and *Muribaculaceae* positively correlated with FSH, E2, IL-10, and BDNF but negatively with T. These findings highlight the role of gut microbiota in modulating inflammation, sex hormone balance, and neurotrophic factor levels. Specifically, PCOS-associated microbiota shifts align with increased inflammation and stress, whereas BL21 intervention promotes beneficial microbiota, supporting reproductive health and neuroprotection.

## DISCUSSION

Studies indicate a close correlation between PCOS and metabolic abnormalities, with the gut playing a pivotal role in nutrient absorption and metabolism. Gut functionality, particularly metabolism, is directly influenced by the gut microbiota. Consequently, probiotics could directly impact metabolic processes through the improvement of gut functionality, thereby affecting diseases related to the endocrine system, such as PCOS. Our study demonstrated the impact of probiotic supplementation on the gut microbiome of PCOS model mice, yet did not delve into the potential connection of nutritional metabolism and PCOS. Observations of this study revealed the multifaceted impacts of BL21 on the alleviation of PCOS symptoms, highlighting its potential in weight management, glucose tolerance, inflammation modulation, sex hormonal balance, and gut microbiota diversification. These outcomes bolster the concept of employing probiotics as a viable PCOS treatment, consistent with BL21’s documented benefits in enhancing glucose regulation, reducing insulin resistance, and optimizing gut microbiome composition in metabolic-related diseases (20, 22, 25). Given the multifactorial nature of PCOS, BL21’s intervention effects suggest its therapeutic promise in PCOS management. Further investigations are essential to fully elucidate these mechanisms and confirm the specific benefits of BL21 in PCOS.

Our study demonstrates that BL21 significantly mitigated weight gain and hinted at enhanced glucose tolerance in a PCOS mouse model, though without marked differences. This reduction in weight gain emphasizes BL21’s role in energy balance and metabolic health, consistent with studies showing probiotics’ impact on gut microbiota and metabolic pathways, including short-chain fatty acid (SCFA) production, which boosts insulin sensitivity and energy expenditure (20, 25–27). The subtle effects on glucose tolerance suggest the complex metabolic influence of probiotics on PCOS, potentially requiring longer durations for observable metabolic corrections, as indicated by prior research (28). BL21’s effect on inflammatory markers highlights its potential in reducing PCOS-associated chronic inflammation, thereby improving symptoms of metabolic syndrome. Key inflammatory markers like IL-6 and TNF-α, central to PCOS pathophysiology and linked to insulin resistance (29, 30), were significantly reduced by BL21, indicating its regulatory effect on inflammation and sex hormone balance. Additionally, the role of probiotics in enhancing gut barrier integrity, thus mitigating endotoxin-induced systemic inflammation and insulin resistance, positions BL21 as a promising agent in addressing metabolic challenges of PCOS (31, 32). Furthermore, BL21 notably increased serum BDNF levels in PCOS mice, suggesting its beneficial impact on neuroendocrine and metabolic functions. Given the importance of BDNF in neural health, energy metabolism, and insulin sensitivity, and its potential in improving ovarian function by mitigating granulosa cell apoptosis (33–35), BL21 may offer a novel avenue for PCOS symptom management through BDNF-mediated pathways. This underscores the multifaceted potential of probiotics like BL21 in PCOS treatment, meriting further investigation to elucidate these mechanisms and validate their therapeutic efficacy.

Besides, our findings indicate that BL21 significantly modulates sex hormone levels and improves ovarian morphology in PCOS mice, highlighting its potential in correcting PCOS-associated hormonal imbalances and enhancing ovarian function. BL21 intervention led to reduced testosterone and LH levels and increased FSH and E2 levels, potentially easing PCOS symptoms like menstrual irregularities and ovulation issues. This sex hormonal regulation aligns with evidence that probiotics can influence hormonal balance through gut microbiota modifications (19). Moreover, BL21 improved ovarian morphology, decreasing atretic follicles and increasing antral and preovulatory follicles, suggesting its role in supporting reproductive function by promoting normal follicular development. This supports the notion that gut microbiota alterations affect ovarian function, with probiotics potentially improving PCOS reproductive symptoms via the gut–ovary axis (36). Considering the impact of BL21 on inflammatory markers, its positive effects on ovarian morphology may also relate to its anti-inflammatory properties. Since PCOS is often marked by chronic inflammation that can impair ovarian function and follicle development, BL21’s modulation of inflammation could foster healthier follicular maturation by reducing ovarian inflammation (37).

Claudins and occludins, crucial components of tight junctions in intestinal epithelial cells, play a vital role in preserving the gut barrier’s integrity, essential for gut health by regulating the selective passage of substances and preventing the entry of pathogens (38, 39). Their function is intricately linked with the gut microbiota, inflammation, and nutritional metabolism. A balanced gut microbiota supports tight junction integrity, whereas dysbiosis can compromise it, increasing gut permeability and promoting inflammation. Similarly, inflammatory states can diminish claudin and occludin expression, disrupting tight junctions and exacerbating gut permeability. Nutritional metabolism also interacts with tight junction integrity, where malabsorption due to disrupted tight junctions can affect the body’s metabolic state, and excessive intake of fats or sugars can further impair these junctions. Our previous study revealed that androgen regulates the expression of connexin 43 to regulate the gap junction between germ cells and Sertoli cells (40). Interestingly, androgen disorder could damage the blood–testis barrier through disturbance of ZO-1, claudin-1, and occludin (41), as well. These observations suggest a close connection of androgen level and cell junctions. Thus, the impact of androgen disorder and gut function is worthy to be delved into in future studies, especially the potential impact on the expression of tight junction proteins and altering gut permeability, to fully understand these mechanisms and their implications for gut health.

**Fig 8:**
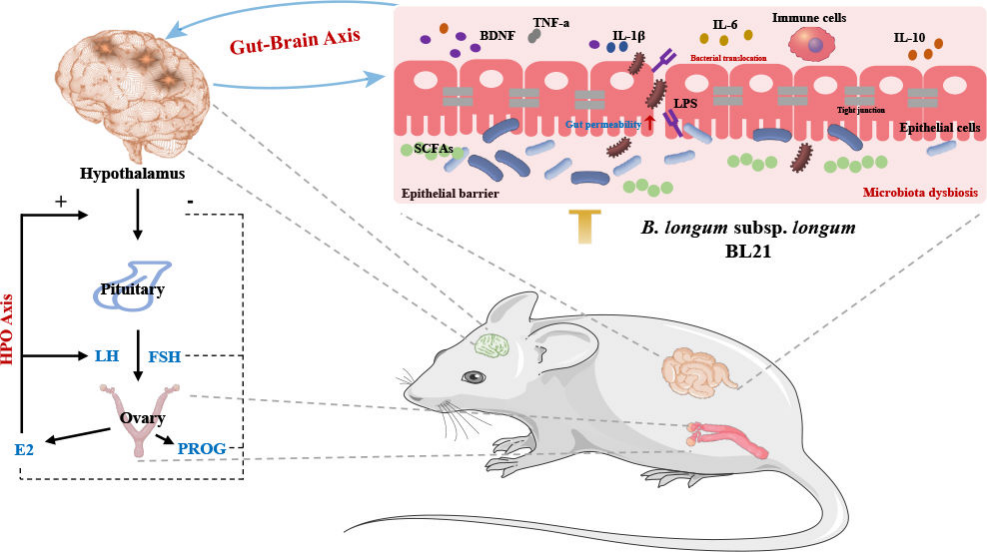
Potential mechanisms of *Bifidobacterium longum* subsp. *longum* BL21 in mitigating PCOS. This schematic illustrates BL21’s potential mechanisms in mitigating PCOS through modulation of the gut–brain and hypothalamic–pituitary–ovarian (HPO) axis, influencing neuroendocrine balance, reducing inflammation, and normalizing sex hormonal profiles.

Furthermore, our analysis highlights BL21’s role in modulating the gut microbiota and its association with inflammatory markers, BDNF, and sex hormones, suggesting potential mechanisms for PCOS symptom alleviation (Fig. 8). BL21 intervention was found to significantly enhance the diversity and alter the composition of the gut microbiome, pivotal for ameliorating PCOS-associated metabolic and endocrine dysfunctions. The gut microbiome, often termed the body’s “second genome,” profoundly influences health, with its imbalance in PCOS patients exacerbating symptoms via inflammation, hormone metabolism, and energy balance pathways (42, 43). Specifically, BL21’s promotion of beneficial genera such as *Bifidobacterium*, *Lactobacillus*, and *Butyricicoccus* suggests its potential in fostering beneficial bacterial colonization and restoring gut ecological balance. This restoration is crucial for combating PCOS pathology through improved gut barrier function, reduced inflammation, and optimized hormone metabolism (44). Notably, *Butyricicoccus* and *Lactobacillus* are linked to enhanced insulin sensitivity and reduced blood sugar levels (25), indicating BL21’s beneficial impact on PCOS metabolic health. Chronic inflammation holds a key position in the pathology of PCOS (45), with studies showing probiotics like *Bifidobacterium* and *Lactobacillus* effectively reducing inflammation via SCFA production, such as butyrate, which inhibits inflammatory cells and markers (46, 47). Thus, BL21’s adjustment of the gut microbiome composition, especially increasing acid-producing bacteria, plays a role in mitigating PCOS-associated chronic inflammation. Moreover, PCOS treatment-induced microbial changes, like those in *Alloprevotella* and *Klebsiella*, correlate with pro-inflammatory factors and stress markers, underscoring the gut microbiome’s regulatory role in host inflammatory responses. The gut microbiome also engages in hormone metabolism and regulation, with BL21 potentially influencing hormonal balance in PCOS by altering the microbial composition. For instance, modulation of the estrogen cycle by certain microbes can impact estrogen levels and activity, crucial for managing estrogen-dependent PCOS symptoms (29). Our findings reveal BL21’s significant effect on the gut microbiome composition related to sex hormones, increasing genera correlated with FSH and E2, such as *Bifidobacterium*, *Bacteroides*, and *Muribaculaceae*. This suggests the gut microbiome’s involvement in hormonal regulation in PCOS, highlighting the strategy of modulating microbial composition to treat PCOS by improving hormonal imbalances. Interestingly, a positive correlation between the gut microbiome and BDNF was discovered, offering new insights into the gut–brain axis’s role in PCOS (48). Given the importance of BDNF in neural development, cognitive function, and emotional regulation, its modulation through the gut microbiome presents a novel therapeutic approach for enhancing neuroprotection and emotional health in PCOS patients.

However, in this study, the estrous cycles in PCOS model mice were not detected, due to the concerns about stress-induced confounding effects from continuous vaginal smear testing. Instead, we relied on multiple established PCOS indicators, including follicle counts, fertility outcomes, hormone levels, and molecular markers. We acknowledge that the absence of estrous cycle analysis represents a limitation of clinic potential. Estrous cycles are closely associated with reproductive function and treatment efficacy in PCOS models, and their omission constrains our ability to fully assess the drug’s impact on reproductive function and hypothalamic–pituitary–ovarian axis regulation. Additionally, estrous cycle data would have enhanced the translational relevance of our findings, given the parallels between mouse estrous cycling and human menstrual patterns in PCOS. Future studies should incorporate continuous monitoring of estrous cycles to provide more comprehensive evidence for therapeutic effects and underlying mechanisms, while carefully controlling for potential stress-related confounders.

This study provides preliminary evidence supporting the therapeutic potential of BL21 in enhancing the diversity of the gut microbiome in a PCOS mouse model, suggesting its prospective benefits for managing PCOS. Furthermore, while this study observes promising correlations between microbiome biomarkers and PCOS phenotypic indicators, additional in-depth investigations are required to establish a definitive causal relationship. Future research should not only explore the variability in responses to probiotics and evaluate their long-term effects and safety profiles but also extend these findings to human clinical trials. It is crucial to investigate various probiotic strains and their interactions within the gut–brain–ovary axis to fully elucidate their roles in PCOS management. This work lays the foundational groundwork for potentially innovative therapeutic strategies and highlights the importance of extensive research and clinical trials to optimize the utilization of probiotics in the treatment of PCOS.

### Conclusion

In conclusion, this study demonstrates the potential of BL21 in mitigating PCOS symptoms through metabolic regulation, inflammation reduction, and neuroprotection. BL21 significantly moderated weight gain, improved glucose tolerance, and adjusted inflammatory and sex hormone levels in a DHT-induced PCOS mouse model. Furthermore, it enhanced gut microbiota diversity, particularly increasing the abundance of beneficial genera associated with positive health outcomes. These findings suggest BL21’s multifaceted role in addressing PCOS, highlighting the importance of the gut microbiome in its therapeutic strategy. Future research should focus on elucidating BL21’s mechanisms and validating its clinical efficacy, aiming to offer more effective PCOS management approaches.

## MATERIALS AND METHODS

### Preparation of strain BL21

Strain BL21 (CGMCC No. 10452), supplied by Wecare Probiotics Co., Ltd., was cultured in strict aseptic conditions using modified MRS medium enriched with 0.1% L-cysteine to promote growth (22). It was incubated anaerobically at 37°C for 22–24 hours and then centrifuged at 6,000 × *g* for 8 minutes at 4°C for cell harvesting (21). The harvested cells were resuspended in sterile water to a concentration of 1 × 10^10^ CFU/mL and stored at 4°C. For consistent viability, a new BL21 suspension was prepared weekly and given to mice at a daily dose of 1 × 10^9^ CFU/mL.

### Prenatal androgen-induced PCOS model and intervention with BL21

This study, conducted at the Stem Cell Research and Translation Center, Nanjing Agricultural University, complied with the EU Guidelines for Laboratory Animal Protection (Directive 2010/63/EU) and was approved by the university’s Animal Ethics Committee. Housed under a 12-hour light/dark cycle, 7-week-old female ICR mice were bred with males, with pregnancy confirmed by vaginal plug detection, marking embryonic day 0.5 (49). Pregnant mice were divided into control (CTL), PCOS model (PCOS), and BL21 intervention (BL21) groups. On gestation days 16–18, PCOS and BL21 groups received 250 µg/100 µL DHT subcutaneously to induce a PCOS model (Fig. 1A). This dosage and administration method were selected based on prior research that demonstrated its effectiveness in reliably mimicking the endocrine and metabolic disturbances characteristic of PCOS, such as hyperandrogenism and insulin resistance (49, 50). Female offsprings (*n* = 8 per group) were studied postnatally. CTL and PCOS groups received purified water, while BL21 was given a BL21 suspension daily. Over 8 weeks, the weight and fecal samples of mice were collected, with samples stored at −80°C. At study end, mice were euthanized humanely, with ovarian tissues collected for analysis and histological examination. Orbital blood was collected for serum samples, which were obtained post-centrifugation at 4,000 × *g* for 10 minutes and stored at −80°C for analysis (25).

### Assessment of oral glucose tolerance

The oral glucose tolerance test was conducted on all mice 2 days prior to the completion of the study (25). The procedure began with mice undergoing a 12-hour fasting period to establish baseline blood glucose levels, which were recorded as the initial measurement at time 0. Subsequently, a glucose solution was administered orally at a dosage of 2 g/kg body weight. Blood samples were then collected from the tail vein at intervals of 30, 60, 90, and 120 minutes post-administration. Blood glucose concentrations were determined using an Accu-Chek blood glucose meter (Roche Applied Science). The obtained glucose values were plotted to generate a time-response curve, and the area under the curve (AUC) was calculated to evaluate oral glucose tolerance.

### Assessment of serum inflammatory markers and sex hormone levels

To evaluate the inflammatory status and endocrine function of the experimental animals, enzyme-linked immunosorbent assay (ELISA) kits (MEIMIAN, Yancheng, Jiangsu, China) were employed for the quantification of serum inflammatory factors and sex hormones. This included the measurement of a range of pro-inflammatory markers such as tumor necrosis factor-alpha (TNF-α), IL-6, interleukin-1 beta (IL-1β), and lipopolysaccharide (LPS), along with anti-inflammatory factors like IL-10 and brain-derived neurotrophic factor (BDNF). Additionally, the levels of sex hormones, including luteinizing hormone (LH), follicle-stimulating hormone (FSH), testosterone (T), estradiol (E2), and progesterone (PROG), were determined. All assays were conducted in strict accordance with the manufacturer’s instructions.

### Histopathological examination of mouse ovarian tissues

Upon culmination of the intervention, mice were anesthetized and euthanized, followed by the prompt collection of various tissue samples, including the ovaries, liver, spleen, pancreas, lungs, and kidneys. These specimens were promptly fixed in a 4% formaldehyde solution, then embedded in paraffin, and sectioned into 5-µm-thick slices. Following this, they were stained with hematoxylin and eosin (51).

### Follicle classification

Total numbers of antral follicles (oocytes surrounded with more than five layers of granulosa cells contain an area of follicular fluid), preovulatory follicles (possess a single large antrum and an oocyte surrounded by cumulus cells at the end of a stalk of mural granulosa cells), and atretic cyst-like follicles (large fluid-filled cyst with an attenuated granulosa cell layer, dispersed theca cell layer, and an oocyte lacking connection with the granulosa cells).

### Follicle and granulosa cell separation

Forty-eight hours prior to the experiment, 10 IU of pregnant mare serum gonadotropin (PMSG) was administered intraperitoneally into the mice. The oocytes and granulosa cells were isolated using a two-step enzymatic and mechanical dissociation process. Initially, the ovarian tissue structure was disrupted with 0.1% collagenase, followed by the addition of 0.3 mg/mL hyaluronidase to facilitate the separation of cumulus–oocyte complexes. Oocytes were manually selected from these complexes under a stereomicroscope. Granulosa cells were subsequently extracted from the remaining follicular fluid and tissue remnants, with a high degree of purity ensured through meticulous pipetting under controlled conditions.

### Reverse transcription polymerase chain reaction (RT-PCR) for mouse ovarian tissue

Mouse ovarian tissues were homogenized in TRNzol Universal reagent for RNA extraction, followed by centrifugation for phase separation and RNA precipitation using chloroform and isopropanol (52). Extracted RNA was washed with 75% ethanol, air-dried, dissolved in RNase-Free ddH_2_O, and stored at 4°C to prevent degradation. Reverse transcription was performed using HiScript III All-in-one RT SuperMix (Vazyme R333-01), with all steps conducted on ice to ensure RNA integrity. See Tables S1 and S2 for detailed parameters.

### Fecal microbiota DNA extraction and 16S rRNA gene sequencing analysis

The gut microbiota in fecal samples was assessed using 16S rRNA gene sequencing, followed by bioinformatics analysis (22). Initially, microbial DNA was extracted from the samples, and the V3–V4 region of the 16S rRNA gene was amplified using PCR with primers 341F (5′-CCTACGGGNGGCWGCAG-3′) and 805R (5′-GACTACHVGGGTATCTAATCC-3′). The PCR protocol included an initial denaturation at 95°C for 3 minutes, followed by denaturation at 94°C for 30 seconds, annealing at 58°C for 30 seconds, extension at 72°C for 45 seconds, and a final extension at 72°C for 5 minutes. The amplified products were then purified using a gel extraction kit (Qiagen, Germany) to ensure the removal of unspecific products and primer dimers. The purified amplicons were subjected to paired-end sequencing on the Illumina MiSeq platform (Illumina, San Diego, USA), with a 2*300 bp configuration to maximize read overlap and accuracy in assembling the amplicons. Raw sequencing data underwent initial processing with USEARCH software, where sequences below a Q20 quality threshold and chimeric sequences were removed. Operational taxonomic units (OTUs) were clustered at a 97% similarity threshold using UPARSE software (version 7.1, http://drive5.com/uparse/), and taxonomic classification was performed with the RDP Classifier (version 2.12, https://bioweb.pasteur.fr/packages/pack@rdp_classifier@2.12) against the RDP database (Release 11.5), applying a confidence threshold of 80%. Bioinformatics analyses, including α diversity, β diversity, principal coordinates analysis (PCoA), and permutational multivariate analysis of variance (PERMANOVA), were conducted using QIIME software (Version 1.9.1). Differential microbial communities among samples were identified using linear discriminant analysis effect size (LEfSe) (53).

### Statistical analysis

Statistical analyses were conducted using R Studio. Data normality was confirmed using the Kolmogorov–Smirnov test or transformed via Blom’s method. For assessing the repeatability and robustness of our experiments, each experimental condition was repeated three times, and each group included independent samples from eight mice to ensure adequate statistical power and minimize potential bias. Multiple group comparisons were performed using the nonparametric Kruskal–Wallis test, while the Mann–Whitney U test was applied for pairwise comparisons, with a significance threshold set at *P* < 0.05. Microbial community structure was analyzed using QIIME software and PCoA based on UniFrac distances, with significance testing conducted using the adonis2 function in the vegan package (54). Community phylogenetic relationships were examined using Statistical Analysis of Metagenomic Profiles (STAMP) and LEfSe analyses. Spearman’s rank correlation tests were employed to investigate associations between serum inflammatory factors, sex hormone levels, and fecal bacterial genera. All analyses were completed in R 4.2.3 (55).

## Data Availability

The data presented in this study are available within the article. The 16S rRNA gene sequence of rat fecal matter has been submitted to the NCBI genome database under PRJNA1080066.

## SUPPLEMENTAL MATERIAL

Supplemental tables msphere.00887-24-s0001.xlsx Tables S1 and S2.

ASM does not own the copyrights to Supplemental Material that may be linked to, or accessed through, an article. The authors have granted ASM a non-exclusive, world-wide license to publish the Supplemental Material files. Please contact the corresponding author directly for reuse.
